# (2,2′-Bipyridine-κ^2^ *N*,*N*′)bis­(*N*-ethyl-*N*-phenyl­dithio­carbamato-κ^2^ *S*,*S*′)cadmium(II) chloro­form solvate

**DOI:** 10.1107/S1600536809041294

**Published:** 2009-10-17

**Authors:** Ibrahim Baba, Nik Ismail Nik Intan, Bohari M. Yamin, Seik Weng Ng

**Affiliations:** a School of Chemical Sciences, Universiti Kebangsaan Malaysia, 43600 Bangi, Selangor Darul Ehsan, Malaysia; b Department of Chemistry, University of Malaya, 50603 Kuala Lumpur, Malaysia

## Abstract

In the title compound, [Cd(C_9_H_10_NS_2_)_2_(C_10_H_8_N_2_)]·CHCl_3_, the Cd^II^ atom exists in an all-*cis* distorted octa­hedral geometry. Chelation is isobidentate for one dithio­carbamate ligand and anisobidentate for the other. The chloroform solvent mol­ecule is disordered over two positions of equal occupancy.

## Related literature

For the crystal structures of other cadmium dithio­carbamate–2,2′-bipyridine adducts, see: Airoldi *et al.* (1990 ▶); Deng *et al.* (2007 ▶); Ivanchenko *et al.* (2000 ▶).
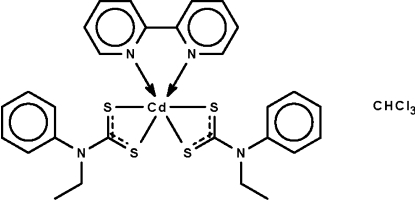


## Experimental

### 

#### Crystal data

[Cd(C_9_H_10_NS_2_)_2_(C_10_H_8_N_2_)]·CHCl_3_
*M* *_r_* = 780.55
Monoclinic, 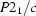

*a* = 7.1911 (6) Å
*b* = 27.752 (3) Å
*c* = 17.1907 (16) Å
β = 99.620 (5)°
*V* = 3382.5 (6) Å^3^
*Z* = 4
Mo *K*α radiation
μ = 1.16 mm^−1^
*T* = 293 K
0.23 × 0.06 × 0.01 mm

#### Data collection

Bruker SMART APEX diffractometer
Absorption correction: multi-scan (*SADABS*; Sheldrick, 1996 ▶) *T*_min_ = 0.777, *T*_max_ = 0.989
18956 measured reflections
5964 independent reflections
4307 reflections with *I* > 2σ(*I*)
*R*_int_ = 0.056

#### Refinement

*R*[*F* ^2^ > 2σ(*F* ^2^)] = 0.070
*wR*(*F* ^2^) = 0.170
*S* = 1.31
5964 reflections
358 parameters
76 restraints
H-atom parameters constrained
Δρ_max_ = 0.85 e Å^−3^
Δρ_min_ = −0.72 e Å^−3^

### 

Data collection: *SMART* (Bruker, 2000 ▶); cell refinement: *SAINT* (Bruker, 2000 ▶); data reduction: *SAINT*; program(s) used to solve structure: *SHELXS97* (Sheldrick, 2008 ▶); program(s) used to refine structure: *SHELXL97* (Sheldrick, 2008 ▶); molecular graphics: *X-SEED* (Barbour, 2001 ▶); software used to prepare material for publication: *publCIF* (Westrip, 2009 ▶).

## Supplementary Material

Crystal structure: contains datablocks global, I. DOI: 10.1107/S1600536809041294/xu2633sup1.cif

Structure factors: contains datablocks I. DOI: 10.1107/S1600536809041294/xu2633Isup2.hkl

Additional supplementary materials:  crystallographic information; 3D view; checkCIF report

## Figures and Tables

**Fig. 1.**
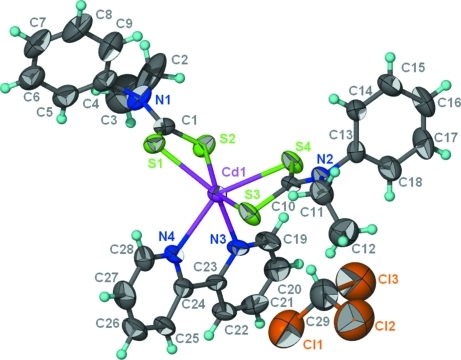
Thermal ellipsoid plot (Barbour, 2001) of Cd(C10H8N2)(C9H10NS2)2.CHCl3 at the 50% probability level. Hydrogen atoms are drawn as spheres of arbitrary radius. The disorder is not shown.

## supplementary crystallographic information

### Experimental

Ethylphenylamine (20 mmol) and carbon disulfide (20 mmol) were dissolved in ethanol (50 ml) at 277 K. Calcium chloride (10 mmol) and 2,2'-bipyridine (10 mmol) dissolved in ethanol (50 mmol) was then added. The white solid that precipitated was collected and recrystallized from an ethanol-chloroform mixture.

### Refinement

As there is some disorder in the ethyl chains of both dithiocarbamate ligands, the C–N distances were tightly restrained to 1.450±0.005 Å and the C–C distances to 1.500±0.005 Å. The chloforom molecule is disordered over two positions; the occupancies were fixed as 0.5 for both components. The six C–Cl distances were restrained to within 0.01 Å of each other as were the Cl···Cl distances. The anisotropic temperature factors of the disordered atoms were restrained to be nearly isotropic.

The phenyl rings were refined as rigid hexagons of 1.39 ° sides.

Carbon-bound H-atoms were placed in calculated positions (C—H 0.93 to 0.97 Å) and were included in the refinement in the riding model approximation, with *U*(H) set to 1.2 to 1.5*U*(C).

### Crystal data

**Table:**

[Cd(C_9_H_10_NS_2_)_2_(C_10_H_8_N_2_)]·CHCl_3_	*F*(000) = 1576
*M**_r_* = 780.55	*D*_x_ = 1.533 Mg m^−^^3^
Monoclinic, *P*2_1_/*c*	Mo *K*α radiation, λ = 0.71073 Å
Hall symbol: -P 2ybc	Cell parameters from 2492 reflections
*a* = 7.1911 (6) Å	θ = 2.5–20.1°
*b* = 27.752 (3) Å	µ = 1.16 mm^−^^1^
*c* = 17.1907 (16) Å	*T* = 293 K
β = 99.620 (5)°	Plate, yellow
*V* = 3382.5 (6) Å^3^	0.23 × 0.06 × 0.01 mm
*Z* = 4	

### Data collection

**Table:**

Bruker SMART APEX diffractometer	5964 independent reflections
Radiation source: fine-focus sealed tube	4307 reflections with *I* > 2σ(*I*)
graphite	*R*_int_ = 0.056
Detector resolution: 8.33 pixels mm^-1^	θ_max_ = 25.0°, θ_min_ = 1.4°
φ and ω scans	*h* = −8→8
Absorption correction: multi-scan (*SADABS*; Sheldrick, 1996)	*k* = −27→33
*T*_min_ = 0.777, *T*_max_ = 0.989	*l* = −20→20
18956 measured reflections	

### Refinement

**Table:**

Refinement on *F*^2^	Primary atom site location: structure-invariant direct methods
Least-squares matrix: full	Secondary atom site location: difference Fourier map
*R*[*F*^2^ > 2σ(*F*^2^)] = 0.070	Hydrogen site location: inferred from neighbouring sites
*wR*(*F*^2^) = 0.170	H-atom parameters constrained
*S* = 1.31	*w* = 1/[σ^2^(*F*_o_^2^) + (0.05*P*)^2^ + 5.0*P*] where *P* = (*F*_o_^2^ + 2*F*_c_^2^)/3
5964 reflections	(Δ/σ)_max_ = 0.001
358 parameters	Δρ_max_ = 0.85 e Å^−^^3^
76 restraints	Δρ_min_ = −0.72 e Å^−^^3^

### Fractional atomic coordinates and isotropic or equivalent isotropic displacement parameters (Å^2^)

**Table:**

	*x*	*y*	*z*	*U*_iso_*/*U*_eq_	Occ. (<1)
Cd1	0.64381 (8)	0.753688 (19)	0.52186 (3)	0.0473 (2)	
Cl1	0.484 (3)	0.6172 (12)	0.3411 (19)	0.163 (3)	0.50
Cl2	0.3864 (15)	0.5394 (4)	0.4309 (8)	0.201 (4)	0.50
Cl3	0.7627 (15)	0.5707 (5)	0.4502 (9)	0.219 (4)	0.50
Cl1'	0.479 (3)	0.6169 (12)	0.3297 (19)	0.163 (3)	0.50
Cl2'	0.2584 (15)	0.5496 (5)	0.3957 (8)	0.201 (4)	0.50
Cl3'	0.6405 (16)	0.5623 (5)	0.4624 (9)	0.219 (4)	0.50
S1	0.6020 (3)	0.84105 (7)	0.57280 (13)	0.0553 (5)	
S2	0.9794 (3)	0.80367 (8)	0.55465 (14)	0.0620 (6)	
S3	0.3644 (3)	0.69545 (9)	0.55197 (13)	0.0648 (6)	
S4	0.7601 (3)	0.68697 (8)	0.63055 (13)	0.0603 (6)	
N1	0.9189 (8)	0.8918 (2)	0.6032 (5)	0.069 (2)	
N2	0.4809 (7)	0.6337 (2)	0.6669 (4)	0.0562 (17)	
N3	0.7617 (8)	0.7162 (2)	0.4173 (3)	0.0520 (16)	
N4	0.4490 (8)	0.7698 (2)	0.3965 (3)	0.0485 (15)	
C1	0.8430 (10)	0.8488 (3)	0.5790 (4)	0.0511 (19)	
C2	1.1238 (10)	0.8984 (4)	0.6258 (9)	0.142 (6)	
H2A	1.1532	0.9172	0.6738	0.170*	
H2B	1.1876	0.8675	0.6337	0.170*	
C3	1.179 (2)	0.9245 (6)	0.5578 (9)	0.190 (8)	
H3A	1.3126	0.9306	0.5680	0.285*	
H3B	1.1126	0.9546	0.5505	0.285*	
H3C	1.1486	0.9053	0.5110	0.285*	
C4	0.8064 (7)	0.93085 (18)	0.6236 (4)	0.066 (2)	
C5	0.7457 (9)	0.9666 (2)	0.5687 (3)	0.082 (3)	
H5	0.7698	0.9639	0.5173	0.098*	
C6	0.6491 (8)	1.0065 (2)	0.5904 (5)	0.089 (3)	
H6	0.6085	1.0304	0.5536	0.107*	
C7	0.6131 (8)	1.0105 (2)	0.6671 (5)	0.096 (4)	
H7	0.5484	1.0372	0.6816	0.115*	
C8	0.6738 (10)	0.9748 (3)	0.7220 (4)	0.106 (4)	
H8	0.6497	0.9775	0.7734	0.127*	
C9	0.7704 (9)	0.9349 (2)	0.7003 (4)	0.092 (3)	
H9	0.8110	0.9110	0.7371	0.110*	
C10	0.5316 (9)	0.6685 (3)	0.6205 (4)	0.0444 (17)	
C11	0.2868 (9)	0.6185 (3)	0.6667 (5)	0.074 (3)	
H11A	0.2024	0.6449	0.6486	0.089*	
H11B	0.2707	0.6105	0.7201	0.089*	
C12	0.2348 (17)	0.5758 (3)	0.6147 (7)	0.109 (4)	
H12A	0.1066	0.5668	0.6163	0.164*	
H12B	0.3168	0.5493	0.6328	0.164*	
H12C	0.2477	0.5837	0.5615	0.164*	
C13	0.6215 (7)	0.61172 (18)	0.7254 (3)	0.0520 (19)	
C14	0.6465 (8)	0.62834 (18)	0.8028 (3)	0.065 (2)	
H14	0.5752	0.6541	0.8160	0.078*	
C15	0.7781 (9)	0.6064 (2)	0.8604 (3)	0.084 (3)	
H15	0.7948	0.6175	0.9121	0.101*	
C16	0.8846 (8)	0.5679 (2)	0.8406 (4)	0.093 (3)	
H16	0.9726	0.5532	0.8792	0.112*	
C17	0.8596 (8)	0.5513 (2)	0.7633 (4)	0.090 (3)	
H17	0.9309	0.5255	0.7501	0.108*	
C18	0.7281 (9)	0.5732 (2)	0.7057 (3)	0.076 (3)	
H18	0.7114	0.5621	0.6540	0.092*	
C19	0.9258 (11)	0.6929 (3)	0.4302 (5)	0.071 (3)	
H19	0.9829	0.6873	0.4821	0.085*	
C20	1.0139 (13)	0.6769 (4)	0.3707 (6)	0.082 (3)	
H20	1.1280	0.6605	0.3822	0.098*	
C21	0.9331 (13)	0.6851 (3)	0.2945 (6)	0.073 (3)	
H21	0.9920	0.6752	0.2530	0.088*	
C22	0.7648 (13)	0.7082 (3)	0.2806 (5)	0.067 (2)	
H22	0.7058	0.7136	0.2290	0.080*	
C23	0.6803 (10)	0.7236 (3)	0.3425 (4)	0.0488 (18)	
C24	0.4952 (10)	0.7492 (3)	0.3318 (4)	0.0512 (18)	
C25	0.3768 (12)	0.7501 (3)	0.2588 (5)	0.064 (2)	
H25	0.4117	0.7350	0.2151	0.077*	
C26	0.2083 (14)	0.7738 (4)	0.2528 (6)	0.080 (3)	
H26	0.1269	0.7751	0.2048	0.096*	
C27	0.1606 (12)	0.7954 (4)	0.3178 (7)	0.081 (3)	
H27	0.0460	0.8114	0.3149	0.098*	
C28	0.2842 (11)	0.7932 (3)	0.3876 (5)	0.063 (2)	
H28	0.2518	0.8089	0.4312	0.076*	
C29	0.5297 (17)	0.5900 (6)	0.4329 (14)	0.117 (6)	0.50
H29	0.5059	0.6125	0.4741	0.141*	0.50
C29'	0.4379 (19)	0.5918 (6)	0.4173 (13)	0.117 (6)	0.50
H29'	0.4021	0.6168	0.4522	0.141*	0.50

### Atomic displacement parameters (Å^2^)

**Table:**

	*U*^11^	*U*^22^	*U*^33^	*U*^12^	*U*^13^	*U*^23^
Cd1	0.0559 (3)	0.0480 (3)	0.0360 (3)	−0.0026 (3)	0.0020 (2)	−0.0005 (3)
Cl1	0.184 (4)	0.141 (3)	0.178 (6)	−0.006 (3)	0.071 (3)	−0.005 (4)
Cl2	0.221 (7)	0.174 (5)	0.217 (7)	−0.039 (6)	0.063 (6)	0.007 (5)
Cl3	0.225 (7)	0.212 (6)	0.208 (6)	0.018 (6)	0.000 (6)	−0.007 (5)
Cl1'	0.184 (4)	0.141 (3)	0.178 (6)	−0.006 (3)	0.071 (3)	−0.005 (4)
Cl2'	0.221 (7)	0.174 (5)	0.217 (7)	−0.039 (6)	0.063 (6)	0.007 (5)
Cl3'	0.225 (7)	0.212 (6)	0.208 (6)	0.018 (6)	0.000 (6)	−0.007 (5)
S1	0.0463 (10)	0.0549 (12)	0.0626 (13)	−0.0009 (9)	0.0029 (9)	−0.0126 (10)
S2	0.0562 (12)	0.0569 (12)	0.0749 (15)	0.0013 (10)	0.0168 (11)	−0.0120 (11)
S3	0.0468 (11)	0.0820 (15)	0.0588 (13)	−0.0022 (11)	−0.0107 (10)	0.0217 (11)
S4	0.0455 (10)	0.0728 (14)	0.0572 (13)	−0.0096 (10)	−0.0072 (9)	0.0204 (11)
N1	0.041 (4)	0.056 (4)	0.113 (6)	−0.005 (3)	0.017 (4)	−0.025 (4)
N2	0.044 (3)	0.064 (4)	0.056 (4)	−0.005 (3)	−0.002 (3)	0.013 (3)
N3	0.050 (4)	0.065 (4)	0.037 (4)	0.001 (3)	−0.003 (3)	−0.010 (3)
N4	0.049 (3)	0.055 (4)	0.039 (4)	0.002 (3)	0.001 (3)	0.004 (3)
C1	0.055 (4)	0.054 (5)	0.044 (4)	−0.004 (4)	0.006 (4)	−0.008 (4)
C2	0.138 (12)	0.066 (7)	0.254 (19)	−0.006 (7)	0.127 (13)	−0.033 (9)
C3	0.109 (11)	0.182 (18)	0.27 (2)	0.035 (12)	0.010 (14)	−0.054 (17)
C4	0.051 (5)	0.059 (5)	0.086 (7)	−0.016 (4)	0.004 (5)	−0.019 (5)
C5	0.066 (6)	0.079 (7)	0.095 (8)	0.006 (5)	−0.005 (5)	−0.028 (6)
C6	0.061 (6)	0.075 (7)	0.120 (10)	0.003 (5)	−0.017 (6)	−0.030 (6)
C7	0.073 (6)	0.061 (6)	0.154 (12)	−0.008 (5)	0.018 (7)	−0.039 (7)
C8	0.114 (9)	0.077 (8)	0.136 (11)	−0.005 (7)	0.050 (8)	−0.044 (8)
C9	0.101 (8)	0.059 (6)	0.123 (10)	−0.005 (6)	0.039 (7)	−0.008 (6)
C10	0.044 (4)	0.054 (4)	0.032 (4)	0.000 (3)	−0.002 (3)	0.000 (3)
C11	0.064 (5)	0.080 (6)	0.076 (7)	−0.003 (5)	0.006 (5)	0.014 (5)
C12	0.114 (9)	0.097 (8)	0.114 (10)	−0.028 (7)	0.012 (8)	−0.003 (7)
C13	0.055 (4)	0.046 (4)	0.057 (5)	−0.001 (4)	0.013 (4)	0.012 (4)
C14	0.082 (6)	0.052 (5)	0.058 (5)	0.003 (4)	−0.001 (5)	0.003 (4)
C15	0.098 (7)	0.085 (7)	0.060 (6)	−0.011 (6)	−0.013 (5)	0.012 (5)
C16	0.079 (7)	0.103 (8)	0.092 (8)	0.018 (6)	−0.004 (6)	0.038 (7)
C17	0.073 (6)	0.096 (8)	0.102 (8)	0.038 (6)	0.017 (6)	0.032 (7)
C18	0.088 (6)	0.087 (7)	0.058 (6)	0.022 (6)	0.023 (5)	0.010 (5)
C19	0.056 (5)	0.099 (7)	0.054 (5)	0.018 (5)	−0.005 (4)	−0.009 (5)
C20	0.058 (5)	0.094 (7)	0.091 (8)	0.021 (5)	0.005 (5)	−0.021 (6)
C21	0.070 (6)	0.085 (7)	0.066 (6)	−0.008 (5)	0.017 (5)	−0.026 (5)
C22	0.085 (6)	0.075 (6)	0.040 (5)	−0.006 (5)	0.008 (4)	−0.005 (4)
C23	0.053 (4)	0.052 (4)	0.039 (4)	−0.013 (4)	−0.001 (4)	−0.004 (3)
C24	0.057 (4)	0.048 (4)	0.047 (4)	−0.013 (4)	0.006 (4)	0.009 (4)
C25	0.067 (5)	0.068 (5)	0.050 (5)	−0.005 (5)	−0.013 (4)	0.007 (4)
C26	0.069 (6)	0.094 (7)	0.068 (7)	−0.010 (6)	−0.018 (5)	0.013 (6)
C27	0.046 (5)	0.084 (7)	0.108 (9)	0.008 (5)	−0.005 (5)	0.017 (6)
C28	0.051 (5)	0.061 (5)	0.077 (6)	0.005 (4)	0.010 (4)	0.012 (4)
C29	0.126 (11)	0.105 (8)	0.121 (9)	0.009 (8)	0.022 (9)	0.010 (7)
C29'	0.126 (11)	0.105 (8)	0.121 (9)	0.009 (8)	0.022 (9)	0.010 (7)

### Geometric parameters (Å, °)

**Table:**

Cd1—N3	2.355 (6)	C8—C9	1.3900
Cd1—N4	2.408 (6)	C8—H8	0.9300
Cd1—S1	2.612 (2)	C9—H9	0.9300
Cd1—S4	2.664 (2)	C11—C12	1.493 (5)
Cd1—S3	2.696 (2)	C11—H11A	0.9700
Cd1—S2	2.759 (2)	C11—H11B	0.9700
Cl1—C29	1.731 (9)	C12—H12A	0.9600
Cl2—C29	1.740 (9)	C12—H12B	0.9600
Cl3—C29	1.737 (9)	C12—H12C	0.9600
Cl1'—C29'	1.730 (9)	C13—C14	1.3900
Cl2'—C29'	1.736 (9)	C13—C18	1.3900
Cl3'—C29'	1.736 (9)	C14—C15	1.3900
S1—C1	1.731 (8)	C14—H14	0.9300
S2—C1	1.686 (8)	C15—C16	1.3900
S3—C10	1.709 (7)	C15—H15	0.9300
S4—C10	1.703 (7)	C16—C17	1.3900
N1—C1	1.350 (9)	C16—H16	0.9300
N1—C4	1.430 (7)	C17—C18	1.3900
N1—C2	1.471 (5)	C17—H17	0.9300
N2—C10	1.341 (9)	C18—H18	0.9300
N2—C13	1.438 (7)	C19—C20	1.363 (12)
N2—C11	1.458 (5)	C19—H19	0.9300
N3—C19	1.332 (10)	C20—C21	1.362 (13)
N3—C23	1.335 (9)	C20—H20	0.9300
N4—C28	1.338 (9)	C21—C22	1.354 (12)
N4—C24	1.341 (10)	C21—H21	0.9300
C2—C3	1.486 (5)	C22—C23	1.379 (11)
C2—H2A	0.9700	C22—H22	0.9300
C2—H2B	0.9700	C23—C24	1.493 (11)
C3—H3A	0.9600	C24—C25	1.394 (11)
C3—H3B	0.9600	C25—C26	1.367 (13)
C3—H3C	0.9600	C25—H25	0.9300
C4—C5	1.3900	C26—C27	1.362 (14)
C4—C9	1.3900	C26—H26	0.9300
C5—C6	1.3900	C27—C28	1.370 (12)
C5—H5	0.9300	C27—H27	0.9300
C6—C7	1.3900	C28—H28	0.9300
C6—H6	0.9300	C29—H29	0.9800
C7—C8	1.3900	C29'—H29'	0.9800
C7—H7	0.9300		
			
N3—Cd1—N4	68.2 (2)	N2—C11—H11B	109.2
N3—Cd1—S1	137.94 (17)	C12—C11—H11B	109.2
N4—Cd1—S1	92.60 (15)	H11A—C11—H11B	107.9
N3—Cd1—S4	96.75 (16)	C11—C12—H12A	109.5
N4—Cd1—S4	145.52 (15)	C11—C12—H12B	109.5
S1—Cd1—S4	116.75 (7)	H12A—C12—H12B	109.5
N3—Cd1—S3	104.88 (16)	C11—C12—H12C	109.5
N4—Cd1—S3	86.69 (15)	H12A—C12—H12C	109.5
S1—Cd1—S3	111.16 (7)	H12B—C12—H12C	109.5
S4—Cd1—S3	66.76 (6)	C14—C13—C18	120.0
N3—Cd1—S2	87.94 (15)	C14—C13—N2	119.5 (5)
N4—Cd1—S2	116.60 (15)	C18—C13—N2	120.5 (5)
S1—Cd1—S2	67.08 (6)	C13—C14—C15	120.0
S4—Cd1—S2	92.56 (7)	C13—C14—H14	120.0
S3—Cd1—S2	156.49 (7)	C15—C14—H14	120.0
C1—S1—Cd1	88.0 (3)	C14—C15—C16	120.0
C1—S2—Cd1	84.2 (3)	C14—C15—H15	120.0
C10—S3—Cd1	86.2 (3)	C16—C15—H15	120.0
C10—S4—Cd1	87.3 (2)	C17—C16—C15	120.0
C1—N1—C4	122.0 (6)	C17—C16—H16	120.0
C1—N1—C2	121.8 (7)	C15—C16—H16	120.0
C4—N1—C2	115.1 (7)	C16—C17—C18	120.0
C10—N2—C13	119.5 (5)	C16—C17—H17	120.0
C10—N2—C11	124.2 (6)	C18—C17—H17	120.0
C13—N2—C11	116.1 (6)	C17—C18—C13	120.0
C19—N3—C23	118.0 (7)	C17—C18—H18	120.0
C19—N3—Cd1	120.6 (5)	C13—C18—H18	120.0
C23—N3—Cd1	120.7 (5)	N3—C19—C20	122.8 (8)
C28—N4—C24	116.8 (7)	N3—C19—H19	118.6
C28—N4—Cd1	124.3 (5)	C20—C19—H19	118.6
C24—N4—Cd1	118.4 (5)	C19—C20—C21	119.4 (8)
N1—C1—S2	120.8 (6)	C19—C20—H20	120.3
N1—C1—S1	118.5 (6)	C21—C20—H20	120.3
S2—C1—S1	120.7 (4)	C22—C21—C20	118.2 (9)
C3—C2—N1	104.0 (11)	C22—C21—H21	120.9
C3—C2—H2A	111.0	C20—C21—H21	120.9
N1—C2—H2A	111.0	C21—C22—C23	120.5 (8)
C3—C2—H2B	111.0	C21—C22—H22	119.8
N1—C2—H2B	111.0	C23—C22—H22	119.8
H2A—C2—H2B	109.0	N3—C23—C22	121.1 (7)
C2—C3—H3A	109.5	N3—C23—C24	115.5 (7)
C2—C3—H3B	109.5	C22—C23—C24	123.4 (7)
H3A—C3—H3B	109.5	N4—C24—C25	122.7 (8)
C2—C3—H3C	109.5	N4—C24—C23	116.1 (6)
H3A—C3—H3C	109.5	C25—C24—C23	121.2 (7)
H3B—C3—H3C	109.5	C26—C25—C24	118.5 (9)
C5—C4—C9	120.0	C26—C25—H25	120.8
C5—C4—N1	120.0 (6)	C24—C25—H25	120.8
C9—C4—N1	119.8 (6)	C27—C26—C25	119.4 (9)
C6—C5—C4	120.0	C27—C26—H26	120.3
C6—C5—H5	120.0	C25—C26—H26	120.3
C4—C5—H5	120.0	C26—C27—C28	118.9 (9)
C5—C6—C7	120.0	C26—C27—H27	120.5
C5—C6—H6	120.0	C28—C27—H27	120.5
C7—C6—H6	120.0	N4—C28—C27	123.7 (9)
C8—C7—C6	120.0	N4—C28—H28	118.2
C8—C7—H7	120.0	C27—C28—H28	118.2
C6—C7—H7	120.0	Cl1—C29—Cl3	108.6 (8)
C9—C8—C7	120.0	Cl1—C29—Cl2	108.2 (7)
C9—C8—H8	120.0	Cl3—C29—Cl2	107.8 (7)
C7—C8—H8	120.0	Cl1—C29—H29	110.7
C8—C9—C4	120.0	Cl3—C29—H29	110.7
C8—C9—H9	120.0	Cl2—C29—H29	110.7
C4—C9—H9	120.0	Cl1'—C29'—Cl2'	108.3 (7)
N2—C10—S4	120.7 (5)	Cl1'—C29'—Cl3'	109.0 (8)
N2—C10—S3	119.7 (5)	Cl2'—C29'—Cl3'	108.5 (7)
S4—C10—S3	119.6 (4)	Cl1'—C29'—H29'	110.3
N2—C11—C12	112.0 (7)	Cl2'—C29'—H29'	110.3
N2—C11—H11A	109.2	Cl3'—C29'—H29'	110.3
C12—C11—H11A	109.2		
			
N3—Cd1—S1—C1	−57.8 (3)	N1—C4—C5—C6	−175.0 (5)
N4—Cd1—S1—C1	−117.5 (3)	C4—C5—C6—C7	0.0
S4—Cd1—S1—C1	81.3 (3)	C5—C6—C7—C8	0.0
S3—Cd1—S1—C1	155.1 (3)	C6—C7—C8—C9	0.0
S2—Cd1—S1—C1	0.4 (3)	C7—C8—C9—C4	0.0
N3—Cd1—S2—C1	144.9 (3)	C5—C4—C9—C8	0.0
N4—Cd1—S2—C1	80.6 (3)	N1—C4—C9—C8	175.0 (5)
S1—Cd1—S2—C1	−0.4 (3)	C13—N2—C10—S4	−0.1 (10)
S4—Cd1—S2—C1	−118.5 (3)	C11—N2—C10—S4	174.8 (6)
S3—Cd1—S2—C1	−91.0 (3)	C13—N2—C10—S3	−178.9 (5)
N3—Cd1—S3—C10	92.9 (3)	C11—N2—C10—S3	−4.0 (10)
N4—Cd1—S3—C10	159.3 (3)	Cd1—S4—C10—N2	−175.7 (6)
S1—Cd1—S3—C10	−109.2 (3)	Cd1—S4—C10—S3	3.1 (4)
S4—Cd1—S3—C10	1.9 (3)	Cd1—S3—C10—N2	175.7 (6)
S2—Cd1—S3—C10	−28.2 (3)	Cd1—S3—C10—S4	−3.1 (4)
N3—Cd1—S4—C10	−105.2 (3)	C10—N2—C11—C12	95.3 (10)
N4—Cd1—S4—C10	−44.4 (4)	C13—N2—C11—C12	−89.6 (9)
S1—Cd1—S4—C10	101.0 (3)	C10—N2—C13—C14	96.2 (7)
S3—Cd1—S4—C10	−1.9 (3)	C11—N2—C13—C14	−79.1 (7)
S2—Cd1—S4—C10	166.6 (3)	C10—N2—C13—C18	−85.7 (7)
N4—Cd1—N3—C19	174.9 (7)	C11—N2—C13—C18	98.9 (7)
S1—Cd1—N3—C19	106.6 (6)	C18—C13—C14—C15	0.0
S4—Cd1—N3—C19	−37.2 (6)	N2—C13—C14—C15	178.1 (5)
S3—Cd1—N3—C19	−104.9 (6)	C13—C14—C15—C16	0.0
S2—Cd1—N3—C19	55.1 (6)	C14—C15—C16—C17	0.0
N4—Cd1—N3—C23	4.6 (5)	C15—C16—C17—C18	0.0
S1—Cd1—N3—C23	−63.6 (6)	C16—C17—C18—C13	0.0
S4—Cd1—N3—C23	152.5 (5)	C14—C13—C18—C17	0.0
S3—Cd1—N3—C23	84.8 (5)	N2—C13—C18—C17	−178.0 (5)
S2—Cd1—N3—C23	−115.2 (5)	C23—N3—C19—C20	0.7 (13)
N3—Cd1—N4—C28	174.0 (6)	Cd1—N3—C19—C20	−169.8 (7)
S1—Cd1—N4—C28	−44.5 (6)	N3—C19—C20—C21	0.5 (15)
S4—Cd1—N4—C28	105.0 (6)	C19—C20—C21—C22	−1.4 (15)
S3—Cd1—N4—C28	66.5 (6)	C20—C21—C22—C23	1.2 (14)
S2—Cd1—N4—C28	−110.1 (6)	C19—N3—C23—C22	−0.9 (11)
N3—Cd1—N4—C24	2.5 (5)	Cd1—N3—C23—C22	169.6 (6)
S1—Cd1—N4—C24	144.0 (5)	C19—N3—C23—C24	179.0 (7)
S4—Cd1—N4—C24	−66.4 (6)	Cd1—N3—C23—C24	−10.4 (8)
S3—Cd1—N4—C24	−104.9 (5)	C21—C22—C23—N3	0.0 (13)
S2—Cd1—N4—C24	78.4 (5)	C21—C22—C23—C24	180.0 (7)
C4—N1—C1—S2	179.5 (6)	C28—N4—C24—C25	−2.2 (11)
C2—N1—C1—S2	−13.4 (13)	Cd1—N4—C24—C25	169.9 (6)
C4—N1—C1—S1	0.0 (11)	C28—N4—C24—C23	179.4 (6)
C2—N1—C1—S1	167.1 (8)	Cd1—N4—C24—C23	−8.5 (8)
Cd1—S2—C1—N1	−178.8 (7)	N3—C23—C24—N4	12.3 (9)
Cd1—S2—C1—S1	0.7 (4)	C22—C23—C24—N4	−167.7 (7)
Cd1—S1—C1—N1	178.8 (7)	N3—C23—C24—C25	−166.1 (7)
Cd1—S1—C1—S2	−0.7 (5)	C22—C23—C24—C25	13.8 (11)
C1—N1—C2—C3	104.8 (12)	N4—C24—C25—C26	1.1 (12)
C4—N1—C2—C3	−87.3 (12)	C23—C24—C25—C26	179.4 (8)
C1—N1—C4—C5	−97.3 (8)	C24—C25—C26—C27	−0.3 (14)
C2—N1—C4—C5	94.8 (9)	C25—C26—C27—C28	0.6 (15)
C1—N1—C4—C9	87.7 (8)	C24—N4—C28—C27	2.5 (12)
C2—N1—C4—C9	−80.2 (9)	Cd1—N4—C28—C27	−169.0 (7)
C9—C4—C5—C6	0.0	C26—C27—C28—N4	−1.8 (14)
